# Contralateral Distal Fibula Plate for Proximal Radius Fracture: A Technical Case Report

**DOI:** 10.7759/cureus.90558

**Published:** 2025-08-20

**Authors:** Jayanth Kumar, Alex Moses, Porter Young, Adam Gitlin

**Affiliations:** 1 Orthopaedic Surgery, University of Florida College of Medicine – Jacksonville, Jacksonville, USA

**Keywords:** gunshot injuries, isolated radius fracture, open reduction and internal fixation with a plate (orif with plate), proximal radius, radius shaft

## Abstract

Proximal radius fractures are injuries that are very difficult to manage, given the complex anatomy and the rarity of the fracture. Typical fixation of the proximal radius involves open reduction and internal fixation. However, this fixation is challenging given the paucity of proximal radius-specific plating systems. In addition, proximal radius fixation constructs reported in the literature have been shown to have issues with fracture alignment. Our case report aims to highlight the utility of stabilizing proximal radius fractures with a contralateral posterolateral distal fibula plate. It involves a 29-year-old male who sustained a highly comminuted, ballistic left proximal radial shaft fracture that underwent successful fixation, fracture union, and return to function utilizing this plating system.

## Introduction

Proximal radius fractures are rare injuries, commonly occurring from axial loading mechanisms or high-energy blunt force mechanisms [1,2]. Fractures involving the metadiaphyseal region of the proximal radius are a particularly uncommon injury. These injuries are challenging to manage due to their rarity, complex osseous anatomy, and the proximity of critical neurovascular structures, which can limit surgical exposure [3,4].

Despite the availability of radius-specific fixation systems, few are well-suited for the metadiaphyseal segment. Given its unique anatomy, there have been many different fixation constructs of the metadiaphysis. Radial head plates are typically too short to span the metadiaphyseal zone, while standard radial shaft plates are too large to achieve stable fixation in the small proximal fragment often present in these injuries. Demetri et al. described alternative strategies using non-anatomy-specific implants, including volar and metadiaphyseal distal radius plates, locking mini-fragment Y- and T-plates with adjunct lag screws, flexible nails, and 3.5 mm limited contact dynamic compression plates [5]. Higashi et al. even utilized a volar distal ulna plate in the fixation of a proximal radius fracture with metadiaphyseal extension [6]. Another key feature in the fixation of these fractures is the contouring of the plate, as Rupasinghe et al. found that contoured plates provided better forearm rotation in proximal radius fractures [7]. These techniques have shown favorable outcomes; however, there has not been a standardized fixation method described. We describe another novel fixation strategy using a contralateral posterolateral distal fibula plate for proximal radius metadiaphyseal fractures. To our knowledge, this technique has not been previously reported.

## Case presentation

**Figure 1:**
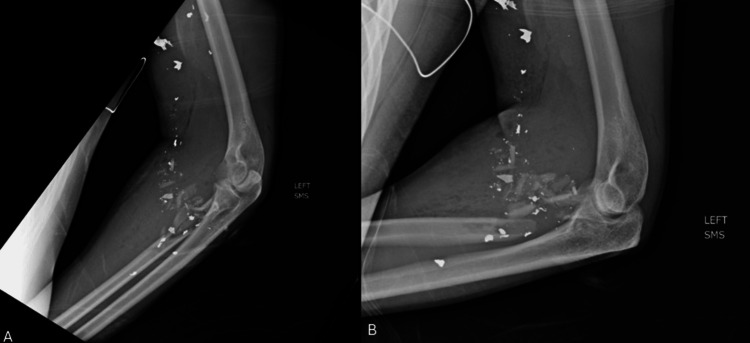
Injury radiographs Anteroposterior (A) and lateral (B) radiographs of the left elbow demonstrating a comminuted proximal radial shaft fracture from a ballistic injury.

**Figure 2:**
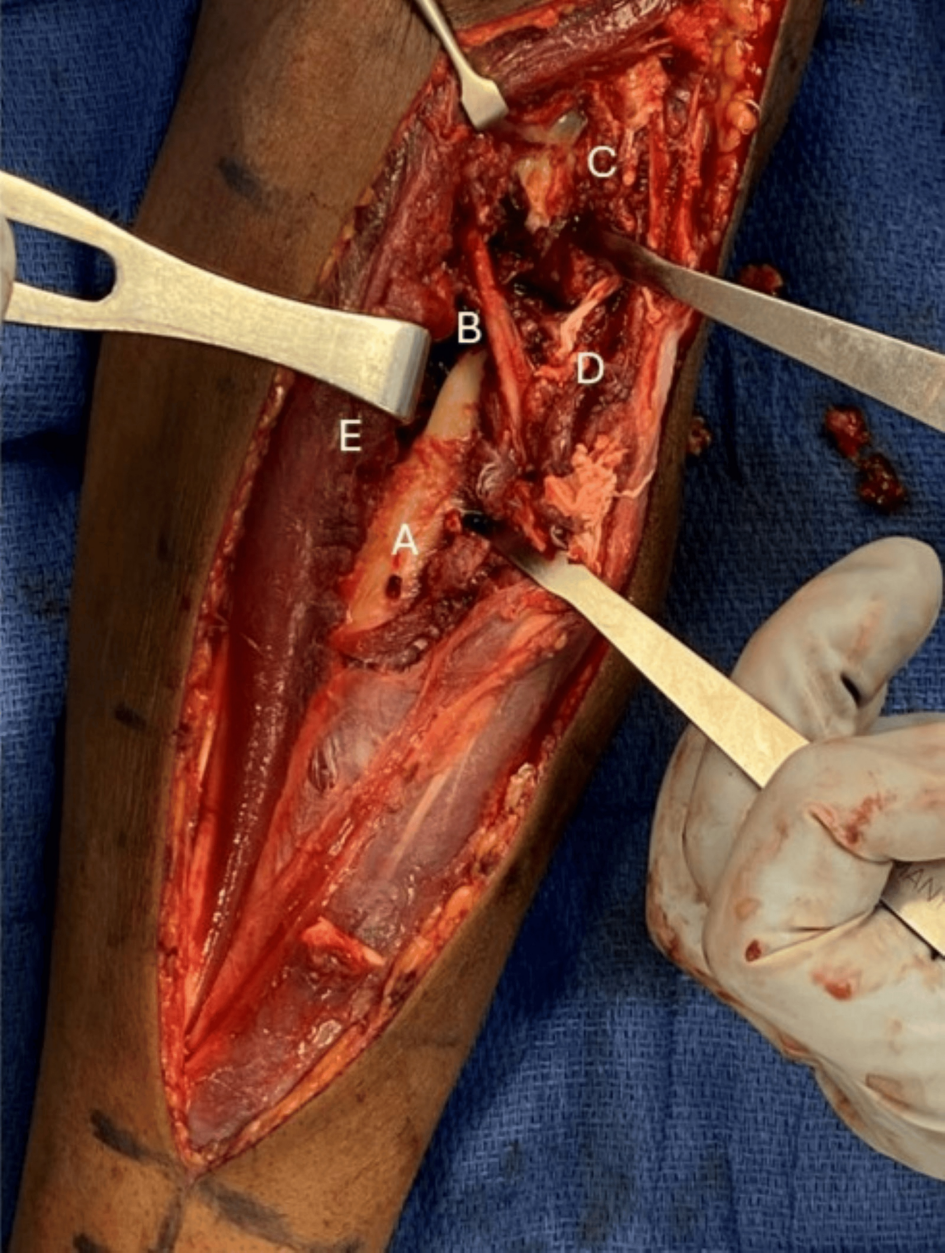
Intraoperative exposure Intraoperative photograph demonstrating the dorsal Thompson approach to the left radial shaft fracture. A: radial shaft, B: Posterior interosseous nerve, C: radial head and neck segment, D: Supinator muscle, E: Extensor carpi radialis brevis muscle

The case involves a 29-year-old male who sustained a comminuted left proximal radius fracture (AO/OTA 2R1C3) secondary to a ballistic injury (Figure 1). Upon arrival at the emergency department, the patient had an open gunshot wound to the proximal forearm and had difficulty with wrist and digit extension, with one out of two sensation to the radial nerve distribution. Otherwise, the patient was neurovascularly intact. The patient was given antibiotics and splinted in the emergency department and was taken to the operating room the same day. The patient was positioned supine on a radiolucent flat-top table with a hand table extension. A sterile tourniquet was applied, and the proximal radius was approached through a dorsal Thompson interval, with careful identification and protection of the posterior interosseous nerve. Following exposure, remnants of the annular ligament were incised in line with the skin incision to facilitate visualization of the radial head (Figure 2).

**Figure 3:**
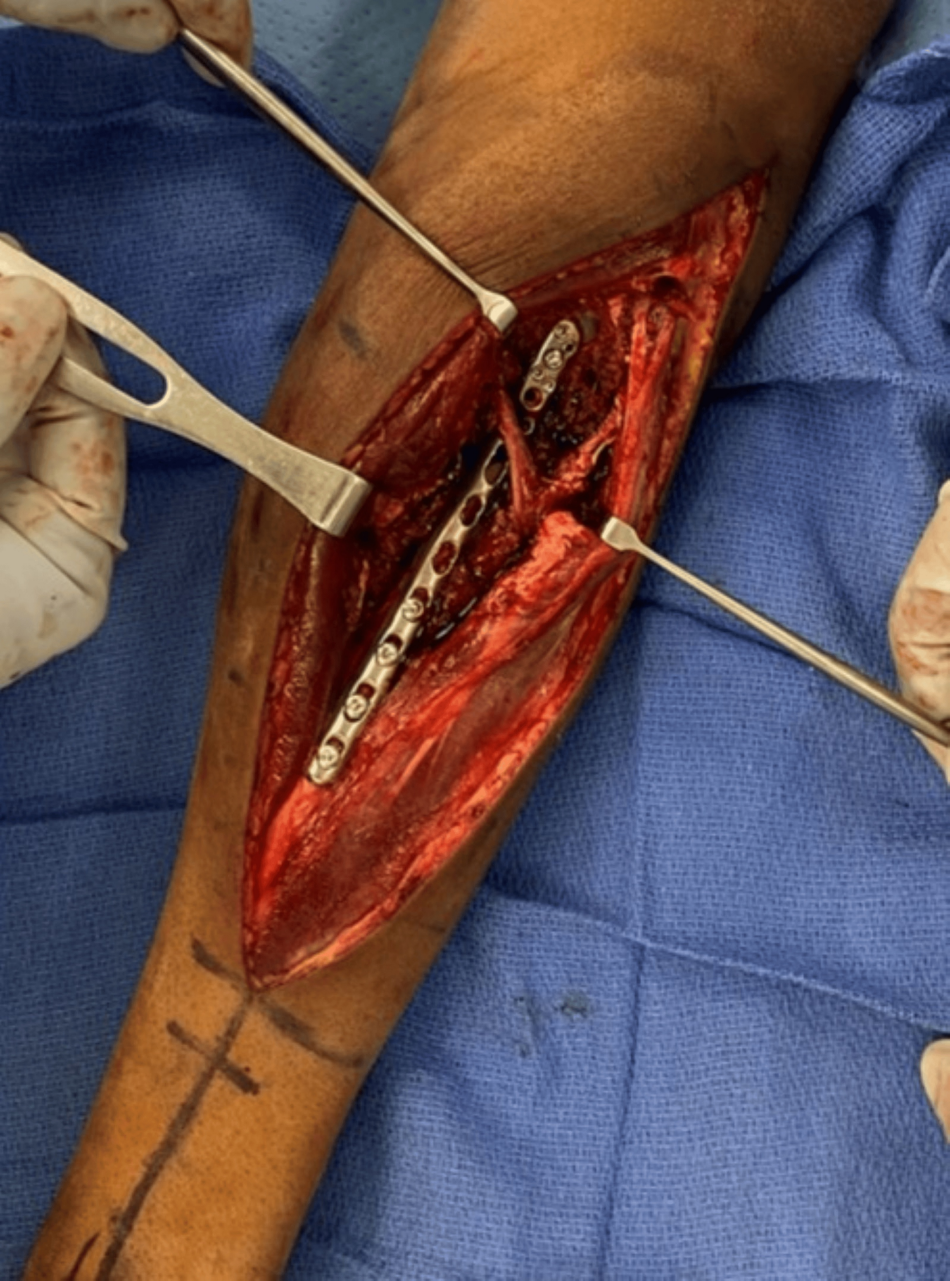
Intraoperative photograph with fixation Intraoperative photograph demonstrating fracture fixation with a right-sided posterolateral distal fibula plate of the patient’s left radial shaft fracture.

**Figure 4:**
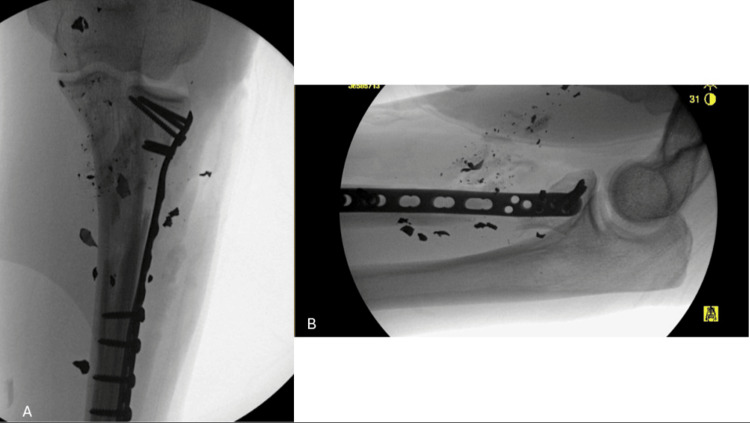
Immediate postoperative fluoroscopic images Anteroposterior (A) and lateral (B) immediate postoperative fluoroscopic images of the left proximal radius fracture status post open reduction internal fixation with a contralateral posterolateral distal fibula plate.

A seven-hole right posterolateral 2.7/3.5 mm Synthes distal fibula locking plate was selected. The plate was contoured to match the proximal radius and positioned with the distal portion of the plate situated within the safe zone of the radial head and neck, allowing for secure fixation of the proximal fragments. This safe zone is a 110-degree arc lying at the posterolateral portion of the radial head where it does not articulate with the ulna. The plate was held onto the bone via serrated reduction clamps and K-wires. Fixation began by placing a 2.7 cortical screw in the radial head, and then a 3.5 cortical screw was placed in the radial shaft. After the position was checked under fluoroscopy, 2.7 locking screws were placed in the proximal holes, and then distal 3.5 cortical screws were placed in the distal plate. Intraoperative assessment confirmed full pronation and supination without evidence of mechanical block prior to closure (Figure 3). Final postoperative X-rays showed an acceptable reduction of the fracture (Figure 4). Postoperatively, the patient was made non-weight-bearing for six weeks and placed in a volar slab splint for one week. This splint was done for soft tissue rest, and the patient was made non-weight-bearing, given the comminuted nature of the fracture. After one week, the splint was removed, and active and passive range of motion of the elbow was initiated.

**Figure 5:**
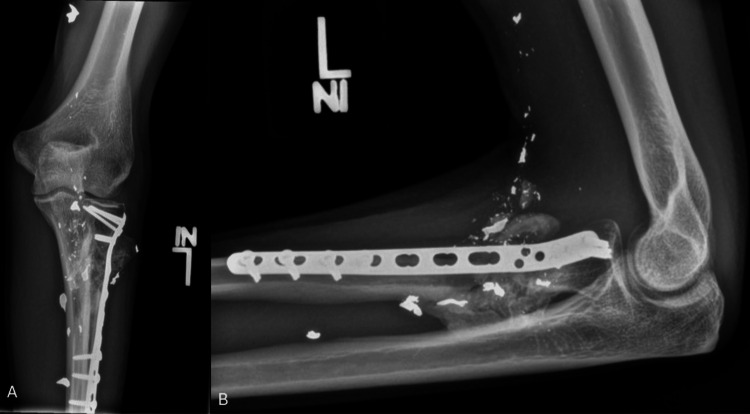
Follow-up radiographs Anteroposterior (A) and lateral (B) radiographs demonstrating a healed proximal radial shaft fracture at 18 months postop fixed with a right-sided posterolateral distal fibula plate.

**Figure 6:**
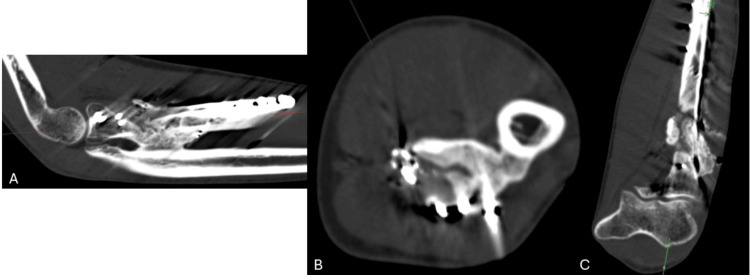
Computerized tomography scan of the left elbow Sagittal (A), axial (B), and coronal (C) slices of the computerized tomography scan of the left elbow obtained at 18 months postoperatively to evaluate for the extent of heterotopic ossification and synostosis formation.

**Figure 7:**
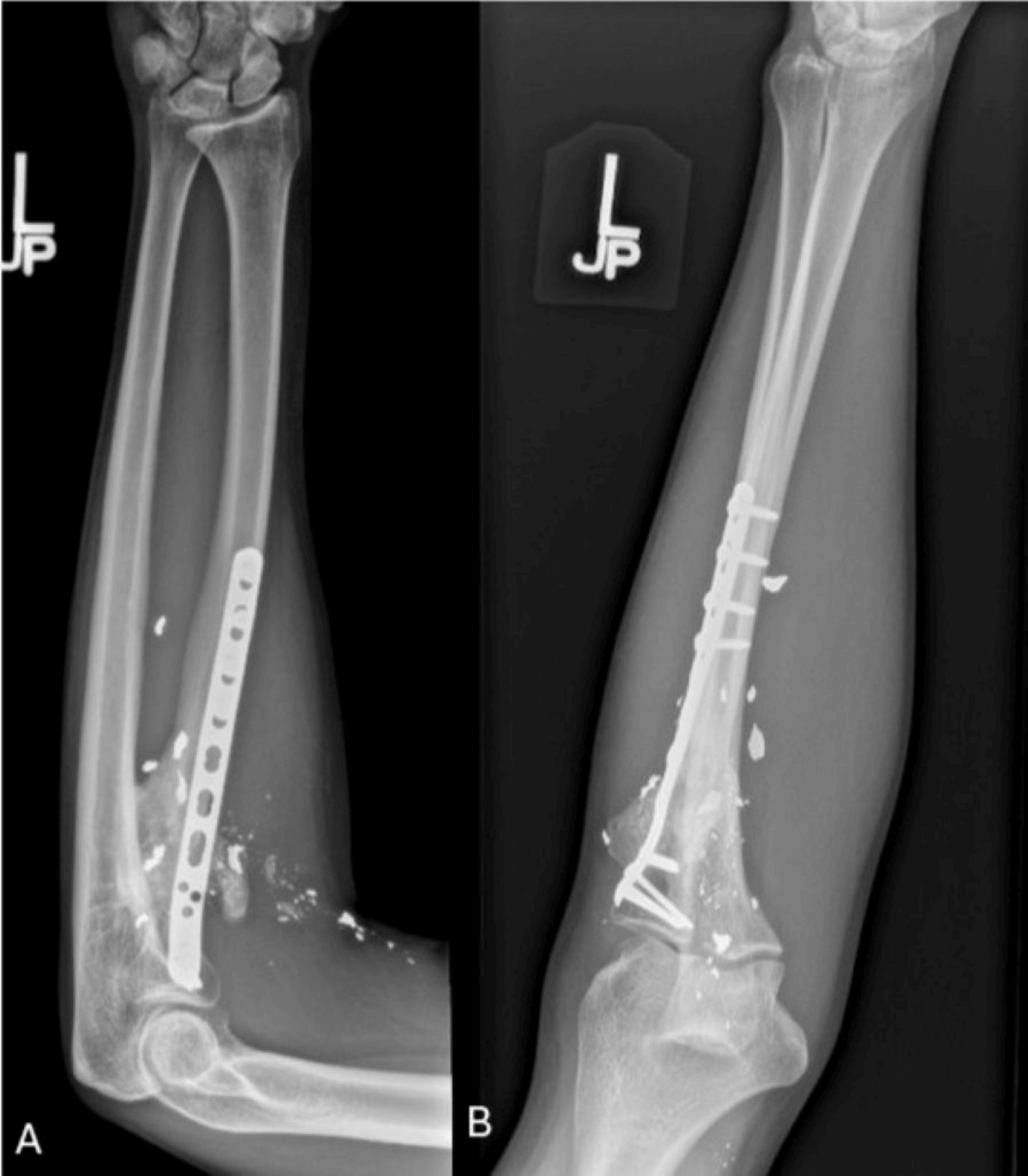
Final left elbow radiographs Anteroposterior (A) and lateral (B) radiographs demonstrating a healed proximal radial shaft fracture at 24 months postop fixed with a right-sided posterolateral distal fibula plate.

Radiographic union was achieved at three months postoperatively. At 18-month follow-up, the patient demonstrated an arc of motion with 0-130° of flexion-extension, 80° of supination, and 15° of pronation. Significant heterotopic ossification was noted; however, there was no evidence of hardware failure or plate impingement (Figure 5). Occupational therapy was initiated to facilitate the range of motion. However, the patient was unable to make improvements in his pronation and supination. Ultimately, a CT scan was ordered to evaluate the mechanical block of motion. The CT showed all hardware intact with heterotopic ossification and synostosis (Figure 6). At the most recent visit, almost two years after the initial surgery, X-rays were obtained, and surgery for heterotopic excision was discussed with the patient, but he declined (Figure 7). Currently, the patient has returned to work as a senior claims examiner and states that he is able to perform his job with appropriate use of his left elbow.

## Discussion

Stable fixation of proximal radius fractures, with the goal of facilitating early postoperative range of motion, requires three key features: (1) sufficient distal plate length to bridge the fracture, (2) multiple proximal fixation points to achieve stable purchase in the small metaphyseal fragment, and (3) a low-profile design to reduce the risk of proximal radioulnar joint impingement. Due to the rarity of these injuries and the complex bony morphology of the proximal radius, few implants are specifically designed for this region in the metadiaphyseal region of the proximal radius. Existing region-specific plates are often too short to span the metadiaphyseal zone, lack adequate proximal fixation options, or fail to conform anatomically, resulting in instability, insufficient purchase, or plate-induced malreduction. Therefore, different region-specific plates have been utilized and contoured in the fixation of these fractures [5].

This case highlights the potential utility of a contralateral posterolateral distal fibula locking plate in addressing these limitations. This Synthes plate offers multiple locking screw trajectories in the proximal fragment and adequate length to span comminuted zones. Furthermore, the anatomic similarity between the distal fibula and proximal radius enables the plate to seat flush with minimal contouring. Both bones are similar anatomically as they have a flare from a narrow shaft to the articular end, and both have grooves through which tendons pass. This anatomic similarity allowed us to utilize the fibula plate and ultimately reduce the risk of implant-related impingement or malreduction.

At 12 weeks, the patient achieved radiographic union without evidence of hardware failure. At 18 months, significant heterotopic ossification and synostosis had developed, accompanied by limitations in pronation. Prophylactic radiation was not considered after the initial surgery but may have been beneficial in this case. Prior studies have shown that ballistic proximal radius fractures are frequently associated with heterotopic ossification and restricted forearm rotation, regardless of fixation method [5]. The intraoperative assessment at the time of surgery confirmed unrestricted pronation, supination, and no mechanical block, suggesting that the plate construct itself was not a contributing factor to the patient’s limited range of motion.

## Conclusions

Proximal radius metadiaphyseal fractures remain a very challenging fracture for orthopedic surgeons to fix, given the difficult anatomy, their infrequency, and the lack of specific implants. Our case illustrates a novel fixation construct for these fractures with the utilization of a contralateral posterolateral distal fibula plate. The low-profile design, enhanced fixation options, and size of these plates offer advantages over reported fixation constructs.
